# Assessment and Psychometric Properties of the 21-Item Depression Anxiety Stress Scale (DASS-21) among Portuguese Higher Education Students during the COVID-19 Pandemic

**DOI:** 10.3390/ejihpe13110177

**Published:** 2023-11-06

**Authors:** Carlos Laranjeira, Ana Querido, Pedro Sousa, Maria Anjos Dixe

**Affiliations:** 1 School of Health Sciences, Polytechnic University of Leiria, Campus 2, Morro do Lena, Alto do Vieiro, Apartado 4137, 2411-901 Leiria, Portugal; ana.querido@ipleiria.pt (A.Q.); maria.dixe@ipleiria.pt (M.A.D.); 2 Centre for Innovative Care and Health Technology (ciTechCare), Rua de Santo André—66–68, Campus 5, Polytechnic University of Leiria, 2410-541 Leiria, Portugal; pmlsousa@esenfc.pt; 3 Comprehensive Health Research Centre (CHRC), University of Évora, 7000-801 Évora, Portugal; 4 Center for Health Technology and Services Research (CINTESIS), NursID, University of Porto, 4200-450 Porto, Portugal; 5 The Health Sciences Research Unit: Nursing (UICISA: E), Nursing School of Coimbra (ESEnfC), Polo A, Av. Bissaya Barreto, 3004-011 Coimbra, Portugal

**Keywords:** reliability, validity, mental health, stress, anxiety, depression, higher education students, COVID-19 pandemic, Portugal

## Abstract

The COVID-19 pandemic has caused substantial disruptions in the lives of higher education students, with detrimental repercussions for academic performance and overall mental health. Therefore, we aimed to evaluate the prevalence of depression, anxiety, and stress symptoms among Portuguese higher education students during the first wave of the coronavirus pandemic and investigate DASS-21’s psychometric characteristics and whether it functions effectively during a pandemic. A convenience sampling procedure was used to recruit 1522 participants (75.1% women and 79.2% undergraduate students) for this cross-sectional research. Participants completed an e-survey created using DASS-21. The results revealed a considerable prevalence of symptoms of depression [≥10] (N = 434, 28.5%), anxiety [≥7] (N = 551, 36.2%), and stress [≥11] (N = 544, 35.7%). Confirmatory factor analysis (CFA) revealed the scale’s three-factor structure, which matched the three DASS-21 subscales. Subsequently, the heterotrait–monotrait (HTMT) correlation ratio evaluated the scale’s discriminant validity, which was relatively good. Cronbach’s alpha measured the internal consistency of the DASS subscales, which was excellent (Cronbach’s α > 0.90). DASS-21 was shown to be a reliable and appropriate measure for assessing students’ mental health. Furthermore, DASS-21 is recommended for use by academics and healthcare professionals in measuring students’ psychological distress. Further validation studies of this scale are needed with larger and more representative samples.

## 1. Introduction 

Depression and anxiety are two of the most difficult mental health challenges faced by young people [1]. The COVID-19 pandemic has heightened this concern [2,3,4]. Several studies have suggested that youths endured more of the pandemic’s tribulations than the general population [5,6]. The prevalence of psychopathological symptoms increased threefold in several countries due to the COVID-19 pandemic’s sanitary crisis [7]. Research shows that the SARS-CoV-2 virus promoted anxiety and depression in COVID-19 survivors, causing direct brain cell invasion, cytokine storms, and neurodegenerative processes [8]. Anosmia, ageusia, and headache are the most frequently reported neurologic symptoms among COVID-19 patients. However, severe adverse events have also been documented, including stroke, loss of consciousness, seizures, cognitive impairment, and increased psychiatric disorders [9,10,11]. Furthermore, the widespread propagation of the coronavirus, its severe effects, and the lockdown measures to reduce infection rates implied restricted social connections and widespread feelings of loneliness, as inferred by increasing internet use (a negative coping strategy) [8]. 

Several international studies documented negative psychological effects among university students because of the COVID-19 pandemic [12,13,14]. Portugal followed this tendency, exhibiting increased rates of anxiety and depression during the pandemic [5,15,16,17,18]. The combination of pandemic-related distress and restrictive preventative measures could have exacerbated pre-existing mental health conditions, giving rise to new symptoms in individuals who had not previously experienced mental health concerns [19]. Accurate psychological tools for screening anxiety and depression symptoms applicable to diverse groups are critical for identifying individuals requiring the attention of mental health professionals [20]. However, the selection of a mental health status assessment instrument may be influenced by several criteria, such as the clinical situation, practitioner type, patient characteristics, and how the findings will be used [21].

The 21-item Depression Anxiety Stress Scale (DASS-21) is a brief self-report scale that measures emotional states of depression, anxiety, and stress (within the last 7 days) [20]. This scale is based on DASS-42, initially developed by Lovibond and Lovibond [22] to cover the full range of core symptoms of emotional distress. Afterward, Antony et al. [23] used seven questions from each original instrument’s subscale to illustrate DASS-21’s reliability and validity. 

DASS-21 is a widely used mental health assessment tool that is simple to use and an accurate tool for research on adults (its intended audience) [24]. This instrument has already been used in research related to virus outbreaks [25]. The three DASS-21 subscales have strong internal consistency and construct validity [26], distinguishing between clinical [27,28,29,30] and nonclinical groups [26,31,32,33]. A tripartite model was proposed to distinguish depression from anxiety and stress [23].

While not a clinical diagnostic measure, “DASS-21 is often used in research and practice in clinical and non-clinical samples to identify individuals in high distress who may be more prone to develop psychopathologies” [34] (p. 4). The original English version of DASS-21 has been assessed in many different countries and 56 languages [35]. DASS-21 has also been used in various situations and age groups, with conflicting findings [36]. There are presently several models, including the three-factor—like in the original DASS-21 research [37,38]—second-order three-factor [39], two-factor [31], one-factor [40], and four-factor [29] models. Several factors may be responsible for differences, including the type of sample (clinical/non-clinical), age of participants, and cultural aspects [41,42].

Considering that the DASS-21 scale has conflicting psychometric properties, we undertook a psychometric investigation to establish the factor structure and reliability of the Portuguese version of DASS-21 when psychological distress becomes ubiquitous, as during the COVID-19 pandemic. Although validated across a variety of demographics, DASS-21 has not been examined with higher education students in Portugal. Therefore, evaluating the use of DASS-21 to assess students’ mental health is essential. To overcome this gap, three specific aims were defined: (a) to evaluate the prevalence of depression, anxiety, and stress symptoms in a sample of Portuguese higher education students during the first wave of the coronavirus pandemic; (b) to determine the reliability, factor structure, and discriminant validity of the DASS-21 scale; and (c) to establish the associations between the DASS-21 subscales and sex, age, marital status, and academic qualifications.

We expected a three-factor model would best match the data. We also anticipated that the DASS-21 scores would vary by age, sex, marital status, and educational background. Finally, we expected significant correlations between the DASS-21 subscales and self-perceptions of physical, mental, and global health.

## 2. Materials and Methods

### 2.1. Study Design

This methodological study targeted higher education students during lockdown in Portugal as a response to the COVID-19 pandemic. It employed cross-sectional data gathered between April and October 2020 (the early phase of the coronavirus pandemic).

### 2.2. Recruitment and Sample

Our participants were Portuguese higher education students enrolled at four educational institutions for the 2020/2021 academic year. These institutions are medium-sized facilities with similar activity profiles located in Portugal’s central region. The sample size was calculated using the formula “sample size = number of items × number of participants”, which is often used in survey development research. We computed “the sample size based on one item and ten participants” [43] (p. 1). Based on the number of DASS-21 items, the minimum intended sample size was 210 answers. 

Eligible participants were adults (over the age of 18) with an education level above high school and the ability to read and comprehend Portuguese. International mobility students were excluded because the e-survey was only developed in Portuguese.

All participants received the necessary information to provide informed consent regarding their participation in the study. They were informed about the scales and the purpose of the research. Given the COVID-19 pandemic, an e-survey was conducted using a convenience sample approach. Google Forms was used to distribute the survey. Participants were contacted using institutional mailing lists sent by the participating institutions’ communication offices and provided a link to access the online questionnaire. Participants could only reply once (multiple responses were blocked), thus reducing the risk of selection bias and anonymizing their identities. We received 1522 valid responses during the survey activation period.

### 2.3. Instruments

The e-survey was composed of three parts:

(1) Basic and academic information, including age, sex, marital status, level of education, and type of educational institution.
(2) Self-perceived physical, mental, and global health, rated on a ten-point scale (1 = poor health to 10 = excellent health), using the previous month as a reference.
(3) Participants’ mental health status, using the Portuguese version of the Depression, Anxiety, and Stress Scale (DASS-21) [38]. Respondents indicated “how much each statement applied to them over the last week on a four-point Likert scale (0 = did not apply to me at all; to 3 = applied to me very much or most of the time)” [44] (p. 3). The scores were added together to provide a total score (0–63) or subtotals for each of the seven subscales (0–21). As recommended for DASS procedures [45], up to one missing item per subscale was permitted for computing the mean score, with missing items replaced by the mean. As DASS-21 is a reduced version of the main scale, which has 42 items, the final score of each subscale must be doubled [45]. The cut-off scores for clinical depression, anxiety, and stress were 10 or more, 7 or more, and 11 or more, respectively [22].

### 2.4. Data Analysis

Frequencies, percentages, medians, ranges, means, and standard deviations (SDs) were used in descriptive data analysis. The data were screened for missing values and normal distribution before analysis. No missing data were detected. The multivariate normality of the variables was assessed using skewness (coefficient of asymmetry) and kurtosis. Severe univariate normality violations to normality were considered when skewness was greater than three and kurtosis greater than seven [46].

Psychometric characteristics of the DASS-21 scale were determined based on fidelity and validity tests [46]. To examine the scale’s reliability, the following criteria were considered: (a) each item’s Pearson correlation with the total scale, wherein correlations greater than 0.20 were desired and other correlations were corrected [47]; (b) the Cronbach’s alpha of all items in each subscale and the scale as a whole, after excluding each item individually. “Cronbach’s alpha allowed us to evaluate the instrument’s internal consistency, which can range from 0 to 1, with higher values indicating better internal consistency” [48] (p. 1). A Cronbach’s alpha value greater than 0.8 indicates good internal consistency, while a value lower than 0.5 is unacceptable [48]. An exploratory factor analysis procedure was carried out, with loadings based on a principal axis factoring with varimax rotation. Factor loadings below 0.5 were suppressed, while item cross-loadings over 0.2 were systematically eliminated, one by one [48]. 

Construct validity was performed using confirmatory factorial analysis (CFA). Chi-square tests are widely used to evaluate how well models fit the data. However, since the χ^2^ statistic is sensitive to sample size, other goodness of fit metrics were applied, namely the goodness of fit index (GFI), the comparative fit index (CFI), the root mean square residual (RMR) index, parsimony-adjusted measures (PNFI and PCFI), the HOELTER index, the expected cross-validation index (ECVI), and Akaike information criteria (AIC) [48]. In this study, two models were tested: Model 1, Lovibond’s original three-factor structure; and Model 2, a three-factor structure of the 21-item DASS based on eliminating items with lower loadings and item–total correlations.

The present study investigated all the correlations among the depression, anxiety, and stress subscales and self-rated perceptions of health to examine the discriminant validity. A heterotrait–monotrait (HTMT) ratio of correlation close to one indicated a lack of discriminant validity. A Pearson correlation coefficient (r) lower than 0.85 indicated a variable’s discriminant validity [49].

An independent-sample *t*-test was used to determine whether there were statistically significant differences between male and female participants in the DASS subscales. The relationship between scale scores and age was calculated using Pearson correlation coefficients. A one-way ANOVA was carried out to examine the relationship between the DASS-21 scale and other sociodemographic characteristics. Post hoc comparisons were performed using the Bonferroni method.

The Statistical Package for Social Sciences (SPSS version 28 for Windows, IBM Corp., Chicago, IL, USA) was used to perform the statistical tests. JASP Version 0.13.1.0 [50] was used for confirmatory factor analysis.

### 2.5. Ethics

The study protocol was authorized by the Ethics Committee of the Polytechnic University of Leiria (CE/IPLEIRIA/22/2020), and the investigation was conducted following the Declaration of Helsinki criteria. Students’ permission was obtained before each e-survey. Participants who agreed to participate completed an informed consent form by ticking the “Yes, I Agree” box, rather than the “No thanks” option, on the online form. To promote truthful replies, the responses were completely anonymized. A unique identification code was assigned to each participating student to associate the questions asked with the corresponding student. Participation was entirely voluntary and unrewarded. 

## 3. Results

### 3.1. Sample Description

The study sample included 1522 individuals, 24.9% male and 75.1% female. The mean age of the participants was 22.85 ± 6.95 (ranging from 18 to 59). Most of the students were single (91.2%; n = 1388), undergraduates (79.2%; n = 1205), and from the public education system (94.1%; n = 1435). Participants reported a mean of 6.51 ± 1.99 for physical health, 6.16 ± 2.14 for mental health, and 6.69 ± 1.79 for global health when asked about their health perceptions during the previous month. 

### 3.2. Base Statistics

Table 1 depicts the base statistics of the DASS-21. Each item’s distribution exhibited positive skewness (ranging from 0.187 to 1.204) and mixed (positive and negative) kurtosis (ranging from −1.081 to 0.377). We assumed that skewness between −2 and +2 and kurtosis between −7 and +7 suggested a normally distributed variable [44]. The DASS-21 scores revealed a considerable prevalence of symptoms of depression [≥10] (N = 434, 28.5%), anxiety [≥7] (N = 551, 36.2%), and stress [≥11] (N = 544, 35.7%).

### 3.3. Reliability of DASS-21 

Cronbach’s alpha was used to calculate internal consistency reliability. The overall alpha was 0.961, and the reliability scores of the three subscales varied from 0.900 (Anxiety) to 0.923 (Stress). Corrected item–total r values varied from 0.536 (item 2) to 0.815 (item 12); factor loadings ranged from 0.569 (item 2) to 0.862 (item 12) (Table 2). Only anxiety item 2, “mouth dryness”, was at the limit of 0.5; however, it was acceptable. The average of the participants’ depression was at the normal level (0–9), as was their anxiety (0–6) and stress (0–10).

### 3.4. Construct Validity

Construct validity was determined using CFA based on the structure of the original DASS-21 scale. Three latent variables, 21 observable variables, and 21 error terms constituted the model framework. The goodness of fit values were reasonable (CFI = 0.931, PNFI = 0.818, PCFI = 0.825, and RMR = 0.038). 

Model refinement was based on modification indices whenever they were adequate from statistical and substantive points of view [51]. Correlation trajectories were established between the errors of items 6 and 18, 8 and 14, 11 and 12, 4 and 7, and 17 and 21. 

Both estimated models were compared by analyzing comparative measures of fit with AIC (Akaike information criteria) and ECVI (the expected cross-validation index). The Model 1 scores were AIC = 1955.215 and ECVI = 1.285. The Model 2 scores were AIC = 1455.204 and ECVI = 0.957. Model 2 was deemed the best-fitting model (Table 3) since lower values suggest a better fit.

**Figure 1:**
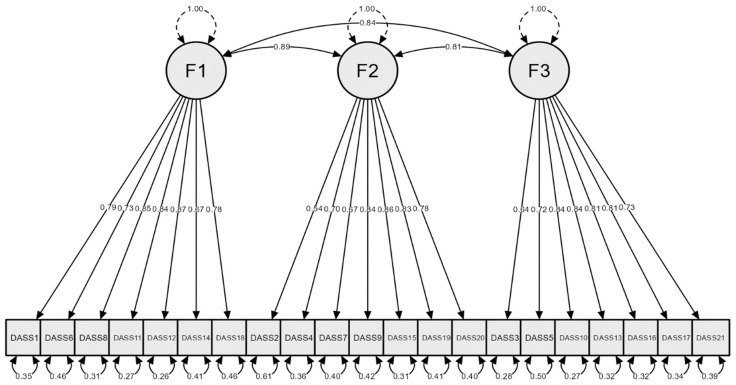
The three-factor model (F1—stress, F2—anxiety, and F3—depression) of the Portuguese DASS-21.

The goodness of fit values in Model 2 yielded a three-factor structure and were adequate (Figure 1), attesting to the factorial validity of the Portuguese version of DASS-21: GFI = 0.919, CFI = 0.952, PNFI = 0.814, PCFI = 0.820, and RMSEA = 0.065. 

### 3.5. Discriminant Validity

Except for the correlation between the anxiety and stress subscales (r = 0.889), the variables all exhibited correlations lower than 0.85. The influence of mental health status, physical health status, and overall health status on the DASS-21 subscale scores was also evaluated. Correlations were statistically significant (*p* < 0.001) and negative, ranging between −0.275 (anxiety–physical health) and −0.539 (stress–mental health) (Table 4).

### 3.6. Associations between DASS-21 Subscales and Sociodemographic Variables

Associations between DASS-21 and sociodemographic variables were assessed by comparing the mean scores across age, sex, marital status, and academic qualifications. The influence of age on the DASS-21 subscale scores demonstrated that correlations were statistically significant (*p* < 0.001) and negative for stress (r = −0.119), anxiety (r = −0.163), and depression (r = −0.151).

The mean scores of the stress and anxiety subscales differed among the sexes. Females reported greater stress (8.909 + 5.716 vs. 6.982 + 5.636; t = 5.708, *p* < 0.001) and anxiety than males (6.086 + 5.618 vs. 4.464 + 4.880; t = 5.392, *p* < 0.001) (Table 5).

Concerning marital status, statistically significant differences (*p* < 0.001) were found among the three DASS-21 subscales (Table 6). A one-way ANOVA with Bonferroni post hoc correction showed significant differences between marital status (between the single and married groups) regarding stress (*p* < 0.001), anxiety (*p* < 0.001), and depression (*p* < 0.001).

Concerning academic qualifications, the one-way ANOVA test revealed statistically significant differences (*p* < 0.05) in the stress and anxiety subscales (Table 7). However, with the Bonferroni post hoc correction, the test only showed significant differences between the Graduation and Other groups regarding stress (*p* = 0.012).

## 4. Discussion

This study endeavored to evaluate the prevalence of anxiety, depression, and stress symptoms, to examine the psychometric properties of the DASS-21 scale, and to determine the associations between the DASS-21 subscales and sex, age, marital status, and academic qualifications in a large sample of Portuguese adults. 

According to Pieh et al. [54], the COVID-19 pandemic and subsequent lockdown measures induced considerable levels of stress among younger people. University students faced significant challenges due to the transition to online study, social isolation from peers, limited access to leisure activities, and the inability to engage in regular student relationships [55]. In our study, the prevalence of mild to extremely severe anxiety, depression, and stress was found in 36.2%, 28.5%, and 35.7% of students, respectively. Similar results were found in previous international studies involving university students during the early stages of the pandemic [56,57]. Previous studies conducted in Portugal reported a lower prevalence of psychopathological symptoms [15,58]. Other studies reported levels of depression and stress higher than our findings, 37.5% and 49.0%, respectively [59]. Maia and Dias [60] also found a notable escalation in psychological distress during the pandemic period in comparison to non-pandemic times. However, the aforementioned data are cross-sectional; hence, a definitive conclusion regarding the causal or direct impact of the pandemic is precluded.

The DASS scale has become an increasingly popular assessment tool for evaluating psychological distress in clinical and non-clinical populations. According to our findings, the Portuguese version of DASS-21 has good psychometric properties. In line with prior research [32,38,61,62,63,64,65,66,67], our results support the DASS-21’s three-factor structure, providing additional evidence of DASS-21’s ability to adjust to many cultures. In addition, these findings will help cross-cultural comparisons [38]. Other studies conducted in Portugal found a different structure [31,68]. In a recent systematic review, the best-fitting models were consistently bifactor models, indicating an underlying unidimensional construct [69].

The reliability of DASS-21 was very good: the reliability of the three subscales ranged from 0.900 (anxiety) to 0.923 (stress). Similar results were found in other studies indicating a three-factor structure [34,38,39,61,63,70], wherein Cronbach’s alpha for depression, anxiety, and stress was ≥0.80 overall, implying good internal consistency reliability. Furthermore, the CFA model’s indexes demonstrated that DASS-21′s three-dimensional structure achieved an adequate fit for the data, and the results were consistent with earlier validation studies [71,72].

Item analysis showed that each scale’s items had good discrimination indices (corrected item–total correlation), indicating that, although the items in each subscale were logically homogeneous, they were not identical or comparable in form and substance. These findings are consistent with those of earlier research [73].

In terms of discriminant validity, the stress and anxiety subscales were substantially correlated, with values higher than those recommended by Kline [49]. These higher correlations may reflect a considerable overlap in the DASS-21 scale content, suggesting a generic concept, such as emotional distress. A similar study found a strong link between these subscales [74]. Discriminant validity was also evaluated between mental health status, physical health status, and overall health status and DASS-21 subscale scores. Correlations were statistically significant and negative for anxiety–physical health and stress–mental health. However, determining validity with other instruments and gold standard criteria, as has been achieved in other studies, is important [26,38,39,61,75].

The associations between personal characteristics and the Portuguese DASS-21 scale scores were also assessed. The examination of differential item functioning (DIF) indicated that the DASS-21 scores varied between the sexes. Our findings revealed that both sexes responded similarly in only three items in the depression subscale and one in the stress subscale. Female participants outperformed males on all DASS-21 subscales, in line with results found in Portugal (depression [38]) and other countries (anxiety and stress) [25,76]. Our results might be explained by the fact that women are more expressive than males and are more sensitive to mental health issues. Unlike our study, other studies found that men exhibit more mental health symptoms [77]. Correlations between age and the DASS-21 subscales were statistically significant and negative for stress, anxiety, and depression, suggesting that DASS-21 is substantially sensitive to age. As people get older, they probably experience fewer mental health issues because their social lives are more restricted than those of younger people. Consequently, they were less negatively impacted by the pandemic than young people. Similar results were obtained via other studies [78].

Concerning academic qualifications, the stress subscale correlated positively with education [26]. In our study, we found statistically significant differences in the stress and anxiety subscales. In terms of marital status, statistically significant differences were found among the three DASS-21 subscales. Our results demonstrated significant differences in stress, anxiety, and depression according to marital status.

Overall, our findings provide evidence that demographic variables seem to influence DASS-21 scores in the Portuguese version. These social determinants have been recognized as psychosocial risk factors that contribute to the occurrence of mental illnesses [79,80]. Nevertheless, more research is required to validate the link between these characteristics and mental disorders. 

Our research included a large sample size and improved the comprehension and use of the scale in a particular historic moment (the COVID-19 pandemic). Nonetheless, some limitations of this research should be addressed when interpreting the findings, such as the manner of administration and the fact that we collected data over the internet. Additional research should be conducted to examine whether there are any significant differences when using alternative modalities of delivery (i.e., clinician interviews and paper-and-pencil questionnaires). DASS-21 was used as a self-assessment tool to detect depression, anxiety, and stress, and no objective clinical examination was performed to validate whether students were in distress. DASS-21 provides a snapshot of a current mental health status, and participants can choose not to disclose their symptoms in order to counteract the prejudice and stigma associated with mental health difficulties [81]. Similarly, self-assessments might be influenced by social desirability bias. The selection of a convenient, non-representative sample of the Portuguese population may impair conclusions and psychometric properties. Our sample included an unbalanced number of female and male participants, as well as a significant age gap between the youngest and oldest students. The present results predominantly reflect female undergraduate students from the public education system. Future research in this area should concentrate on sampling Portuguese percentile norms with a more representative sample in terms of age and gender [31], as well as investigating DASS-21’s longitudinal qualities for test–retest reliability. Further research may also include the study of personality traits and environmental variables that might potentially have a direct influence on the occurrence of depression, anxiety, and stress symptoms among students.

Despite these limitations, our findings have practical implications. While the pandemic no longer constitutes a public health emergency, detecting enduring long-term psychological disorders among students is necessary. Educational institutions and faculty members must prioritize the provision of enhanced mental health support and assistance for students. Online psychological counseling has proven to be especially advantageous during the most critical stages of the COVID-19 pandemic [82,83]. Valid and easily available instruments for psychiatric screening and assessment may considerably assist mental healthcare. DASS-21 is an effective screening tool for stress, anxiety, and depression symptoms. Furthermore, the scale may be used to assess the impacts of health promotion initiatives. The utility of DASS-21 extends beyond the scope of the COVID-19 pandemic and may apply to many other local or global emergencies.

## 5. Conclusions

The present study revealed a higher prevalence of mild-to-extreme symptoms of stress compared to complaints of anxiety and depression, so stress management training might be of high value to support students. Despite significant changes in environmental conditions since the COVID-19 pandemic, the current research found that DASS-21 maintained its reliability and validity, justifying its usage as a mental health screening tool among Portuguese students. The results demonstrated that DASS-21’s three-factor structure has excellent internal consistency. This survey might be expanded to include a broader national representation of students since it only included higher education students in Portugal’s central area.

## Figures and Tables

**Table 1:** Mean scores for DASS-21 and distribution parameters (n = 1522).

DASS-21 Items [22] (p. 179)	Min	Max	M	SD	Mdn	*sk*	*ku*
“1. I found it hard to wind down”	0	3	1.229	0.989	1.0	0.344	−0.917
“2. I was aware of dryness of my mouth”	0	3	0.745	0.952	0.0	1.013	−0.148
“3. I couldn’t seem to experience any positive feeling at all”	0	3	0.702	0.836	0.0	1.005	0.227
“4. I experienced breathing difficulty”	0	3	0.659	0.922	0.0	1.204	0.318
“5. I found it difficult to work up the initiative to do things”	0	3	1.268	1.008	1.0	0.284	−1.013
“6. I tended to over-react to situations”	0	3	1.185	0.996	1.0	0.331	−0.988
“7. I experienced trembling”	0	3	0.637	0.919	0.0	1.241	0.377
“8. I felt that I was using a lot of nervous energy”	0	3	1.351	1.013	1.0	0.187	−1.064
“9. I was worried about situations in which I might panic and make a fool of myself”	0	3	1.046	1.061	1.0	0.540	−1.025
“10. I felt that I had nothing to look forward to”	0	3	0.913	0.987	1.0	0.725	−0.637
“11. I found myself getting agitated”	0	3	1.172	0.985	1.0	0.341	−0.956
“12. I found it difficult to relax”	0	3	1.339	1.007	1.0	0.188	−1.052
“13. I felt down-hearted and blue”	0	3	1.275	1.014	1.0	0.278	−1.032
“14. I was intolerant of anything that kept me from getting on with what I was doing”	0	3	0.924	0.927	1.0	0.617	−0.658
“15. I felt I was close to panic”	0	3	0.806	1.025	0.0	0.957	−0.409
“16. I was unable to become enthusiastic about anything”	0	3	0.927	0.991	1.0	0.730	−0.615
“17. I felt I wasn’t worth much as a person”	0	3	0.804	0.993	0.0	0.945	−0.336
“18. I felt I was rather touchy”	0	3	1.229	1.037	1.0	0.322	−1.081
“19. I was aware of the action of my heart in the absence of physical exertion”	0	3	0.908	1.049	1.0	0.770	−0.743
“20. I felt scared without any good reason”	0	3	0.881	1.001	1.0	0.782	−0.617
“21. I felt that life was meaningless”	0	3	0.685	0.961	0.0	1.202	0.244

Min: minimum; max: maximum; M: mean; SD: standard deviation; Mdn: Median; sk: skewness; ku: kurtosis.

**Table 2:** Descriptive statistics, corrected item–total correlation, factor loadings, and Cronbach’s alpha of the DASS-21 Portuguese version.

	Stress	Anxiety	Depression	Total
*Cronbach’s alpha (α)*	0.923	0.900	0.922	0.961
Mean	8.429	5.682	6.574	-
SD	5.756	5.487	5.613	-
*Corrected item–total correlation*				*Factor loading*
Item 1	0.747			0.800
Item 6	0.722			0.732
Item 8	0.778			0.838
Item 11	0.813			0.852
Item 12	0.815			0.862
Item 14	0.700			0.720
Item 18	0.743			0.756
Item 2		0.536		0.569
Item 4		0.738		0.759
Item 7		0.702		0.725
Item 9		0.730		0.791
Item 15		0.771		0.838
Item 19		0.758		0.789
Item 20		0.713		0.775
Item 3			0.734	0.770
Item 5			0.670	0.710
Item 10			0.808	0.848
Item 13			0.768	0.833
Item 16			0.794	0.821
Item 17			0.788	0.811
Item 21			0.742	0.762

**Table 3:** Summary of the adjustment indices of the DASS-21 Portuguese version.

Measure	Recommended Cutoffs [51,52,53]	DASS-21 Model 1	DASS-21 Model 2
CFI	>0.8	0.931	0.952
GFI	>0.8	0.886	0.919
RMR	<0.05	0.038	0.032
PNFI	>0.5	0.818	0.814
PCFI	>0.5	0.825	0.820
HOELTER (0.1)	>200	191	257
AIC	-	1955.215	1455.204
ECVI	-	1.285	0.957

Comparative fit index (CFI); goodness of fit index (GFI); root mean square residual (RMR) index; parsimony-adjusted measures (PNFI and PCFI); HOELTER index; Akaike information criteria (AIC); and expected cross-validation index (ECVI).

**Table 4:** Pearson correlations between DASS-21 subscales, mental health status, physical health status, and overall health status.

	DASS Stress	DASS Anxiety	DASS Depression
	*r*	*r*	*r*
**DASS stress**	-	0.889 *	0.844 *
**DASS anxiety**	-	-	0.807 *
**DASS depression**	-	-	-
**Mental health perception**	−0.539 *	−0.475 *	−0.551 *
**Physical health perception**	−0.299 *	−0.275 *	−0.287 *
**Overall health perception**	−0.425 *	−0.376 *	−0.417 *

Person correlation coefficient (*r*); *: *p* < 0.001.

**Table 5:** Descriptive statistics of the DASS-21 subscales according to sex.

Subscales	Male (n = 379)		Female (n = 1143)		*t*
	M	SD	M	SD	
**DASS stress**	6.982	5.636	8.909	5.716	5.708 *
**DASS anxiety**	4.464	4.880	6.086	5.618	5.392 *
**DASS depression**	6.161	5.642	6.710	5.600	1.652

M: Mean; SD: standard deviation; independent *t*-test (*t*); *: *p* < 0.001.

**Table 6:** Descriptive statistics of the DASS-21 subscales by marital status.

Subscale	Marital Status	n	M	SD	*F*	*p*
**DASS stress**	Single	1388	8.644	5.750	7.681	***p* < 0.001**
	Married	118	6.059	5.347		
	Divorced	13	7.000	5.859		
	Widowed	3	8.333	2.517		
**DASS anxiety**	Single	1388	5.945	5.529	12.480	***p* < 0.001**
	Married	118	2.864	3.989		
	Divorced	13	3.769	5.904		
	Widowed	3	3.000	2.000		
**DASS depression**	Single	1388	6.860	5.615	14.031	***p* < 0.001**
	Married	118	3.551	4.509		
	Divorced	13	4.154	6.296		
	Widowed	3	3.667	4.726		

M: Mean; SD: standard deviation; one-way ANOVA (F-test).

**Table 7:** Descriptive statistics of the DASS-21 subscales by academic qualification.

Subscale	Academic Qualifications	n	M	SD	*F*	*p*
**DASS stress**	Graduation	1205	8.644	5.708	3.523	**0.015**
	Post-graduation	15	7.333	5.486		
	Master’s degree	154	8.130	5.928		
	Other	148	7.101	5.841		
**DASS anxiety**	Graduation	1205	5.894	5.538	2.919	**0.033**
	Post-graduation	15	4.533	4.704		
	Master’s degree	154	4.831	5.279		
	Other	148	4.959	5.233		
**DASS depression**	Graduation	1205	6.758	5.624	2.133	0.094
	Post-graduation	15	5.533	5.475		
	Master’s degree	154	5.994	5.503		
	Other	148	5.784	5.589		

M: mean; SD: standard deviation; one-way ANOVA (F-test).

## Data Availability

All data generated or analyzed during this study are included in this article.
